# Bioengineered MSC-derived exosomes in skin wound repair and regeneration

**DOI:** 10.3389/fcell.2023.1029671

**Published:** 2023-02-27

**Authors:** Hanxing Zhao, Zhengyong Li, Yixi Wang, Kai Zhou, Hairui Li, Siwei Bi, Yudong Wang, Wenqing Wu, Yeqian Huang, Bo Peng, Jun Tang, Bo Pan, Baoyun Wang, Zhixing Chen, Zhenyu Zhang

**Affiliations:** ^1^ Department of Plastic and Burn Surgery, West China Hospital, Sichuan University, Chengdu, China; ^2^ Department of Plastic Reconstructive and Aesthetic Surgery, West China Tianfu Hospital, Sichuan University, Chengdu, China; ^3^ Plastic Surgery Hospital, Chinese Academy of Medical Sciences and Peking Union Medical College, Beijing, China

**Keywords:** bioengineering, wound healing, skin regeneration, mesenchymal stem cell, exosomes

## Abstract

Refractory skin defects such as pressure ulcers, diabetic ulcers, and vascular ulcers represent a challenge for clinicians and researchers in many aspects. The treatment strategies for wound healing have high cost and limited efficacy. To ease the financial and psychological burden on patients, a more effective therapeutic approach is needed to address the chronic wound. MSC-derived exosomes (MSC-exosomes), the main bioactive extracellular vesicles of the paracrine effect of MSCs, have been proposed as a new potential cell-free approach for wound healing and skin regeneration. The benefits of MSC-exosomes include their ability to promote angiogenesis and cell proliferation, increase collagen production, regulate inflammation, and finally improve tissue regenerative capacity. However, poor targeting and easy removability of MSC-exosomes from the wound are major obstacles to their use in clinical therapy. Thus, the concept of bioengineering technology has been introduced to modify exosomes, enabling higher concentrations and construction of particles of greater stability with specific therapeutic capability. The use of biomaterials to load MSC-exosomes may be a promising strategy to concentrate dose, create the desired therapeutic efficacy, and maintain a sustained release effect. The beneficial role of MSC-exosomes in wound healing is been widely accepted; however, the potential of bioengineering-modified MSC-exosomes remains unclear. In this review, we attempt to summarize the therapeutic applications of modified MSC-exosomes in wound healing and skin regeneration. The challenges and prospects of bioengineered MSC-exosomes are also discussed.

## 1 Introduction

Wounds are an underestimated health problem that lay a great financial burden on patients and healthcare systems (Frykberg and Banks, 2015). As the primary immune barrier against external damage, the skin is the largest organ in the human body that can regulate thermostability and sense extrinsic stimuli (Takeo et al., 2015). The wound healing process is complex and involves inflammation, cell migration, angiogenesis, and granulation tissue formation, and remodelling of the extracellular matrix (ECM) (Cavanagh et al., 2005; Gushiken et al., 2021). A chronic wound, however, is commonly characterized by prolonged inflammation, persistent infection, the formation of microbial biofilms and failure of epithelial cells respond to stimuli (Schultz et al., 2003; Frykberg and Banks, 2015). According to their etiology, the chronic wounds can be classified as diabetic ulcers, pressure ulcers, and vascular ulcers (Nunan et al., 2014). Due to the healing process’s intricacies, treating chronic wounds is still a great challenge for clinicians.

Exosomes derived from mesenchymal stem cells (MSC-exosomes) with regenerative and immunomodulatory functions have been shown to be beneficial in wound healing and to accelerate the process (Casado-Díaz et al., 2020). Compared with conventional treatment methods, MSC-exosome-based therapies have shown higher therapeutic efficacy that play a role throughout all stages of wound healing (Vu et al., 2021). In addition, MSC-exosome-based therapies avoid the risks of immune rejection and tumorigenesis associated with stem cell transplantation (Yin et al., 2019). Therefore, MSC-exosome-based therapy is considered to be a promising therapeutic approach in wound repair and skin regeneration.

In clinical settings, the application of exosome-based therapy may still face some challenges. The challenging task of isolation, purification, and large-scale production of exosomes represents a direct obstacle to the development of exosome-based treatment from bench to bed (Riau et al., 2019). Exosomes may also undergo rapid clearance in the blood circulation or show poor retention at the wound surface (Agrahari et al., 2016; Riau et al., 2019). In addition, safety concerns have limited the clinical applications of MSC-exosomes. The introduction of bioengineering technologies into MSC-exosome-based therapies is expected to address the practical problems mentioned above.

Ideally, modified exosomes could be loaded with luminal cargos or display specific surface molecules, thereby increasing the efficiency of the exosomes and giving them additional therapeutic effects (Riazifar et al., 2017; Wiklander et al., 2019; Salunkhe et al., 2020). Genetic engineering of progenitor cells and direct modification of exosomes are the main engineering strategies for altering exosomes and increasing their regenerative effectiveness (Luan et al., 2017; Shi et al., 2020a). Other strategies such as engineering of hybrid exosomes may also be used in exosome modification (Chen et al., 2020). Furthermore, encapsulating exosomes into hydrogel scaffolding can be used to form a sustained release system or wound dressing (Liang et al., 2019; Bai et al., 2022; Hu et al., 2022). Preclinical studies and clinical trials of chronic wound models have shown promising results, demonstrating that modified MSC-exosomes could promote angiogenesis, fibrogenesis, re-epithelization, and granulation tissue formation, as well as attenuating inflammation (Shi et al., 2020b; Li et al., 2020; Wu et al., 2021a; Xiong et al., 2022). The application of hydrogels may have a synergistic effect in wound closure (Das et al., 2022). Despite a lack of systematic evidence, bioengineered modified MSC-exosomes are increasingly recognized as beneficial to compensate for the limitations of natural exosome-based clinical therapies in wound healing applications.

In this review, we focus on promising bioengineering modifications of MSC-exosomes that enhance their potential therapeutic effectiveness in wound healing and skin regeneration. The applications of biomaterials loaded with modified MSC-exosomes in wound dressing strategies are also addressed. Finally, the challenges and prospects of tissue-engineered MSC-exosomes are discussed. We hope to provide a valuable overview of the role and the mechanisms of modified MSC-exosomes in cutaneous wound healing and elaborate on the potential of applying modified MSC-exosomes in clinical practice.

## 2 Wound healing and regenerative management

The primary molecular regulators of wound healing are proteins and polypeptides including growth factors, cytokines, and chemokines (Das et al., 2015; Guo et al., 2018; Kim et al., 2019; Su et al., 2019). During the past decades, with advances in regenerative medicine, significant efforts have been made to explore solutions to improve the tissue regeneration process, thereby repairing and correcting physiological deficiencies. Such approaches include using growth factors, stem cells, and biomaterials to induce a more effective healing process. However, despite promising therapeutic results in preclinical studies, the clinical applications of these strategies have been limited by concerns including tissue origin, standards for isolation and culture procedures, tumorigenicity, and ethical regulatory restrictions on the use of stem cells.

As stem cells have self-renewal capacity and multi-lineage differentiation potential, they have been extensively explored with respect to their potential applications in treatment of chronic wounds (Donati and Watt, 2015; Chen et al., 2016). However, the immunogenicity and tumorigenesis of stem cells are critical factors affecting their therapeutic function. Owing to their comparatively low immunogenicity, MSCs represent a promising source for cell-based therapies for wound healing (Lee et al., 2014). Accumulating evidence demonstrates that the multi-lineage differentiation of MSCs and their secretion of bioactive factors have potential therapeutic value in the treatment of various diseases (Pittenger et al., 1999; Zhou et al., 2021). However, despite promising results in preclinical studies and clinical trials, MSCs fall short of expectations in clinical settings.

The main limiting factors in the therapeutic use of MSCs are their heterogeneity and limited homing capacity in damaged tissue (Ullah et al., 2019; Levy et al., 2020). On the one hand, MSCs may undergo senescence during extended culture (Fang et al., 2018; Tabibzadeh, 2022). On the other hand, the transplantation of MSCs could induce tumorigenic scenarios. The most critical issue is that the preparation process of MSCs remains experimentally challenging. It is also difficult to reproduce the therapeutic potency of an MSC.

Previous research has attributed the therapeutic effects of MSC-based therapies to the multi-lineage differentiation and engrafting capacities of the MSCs. The current view is that the release of extracellular vesicles (EVs) through paracrine mechanisms is the major mediator of their therapeutic activities (Lener et al., 2015; Li et al., 2018a; Zhang et al., 2019a). Various *in vitro* studies and preclinical disease models have reported that EVs isolated from MSCs cultures display therapeutic activities that equivalent to those of MSCs (Giebel et al., 2017). Therefore, the secretome from MSCs has attracted much attention for its potential use in tissue repair and regeneration.

## 3 EVs from MSCs

### 3.1 EV biogenesis

The term “extracellular vesicles” refers to a group of cell-derived lipid bilayer membranous structures containing transmembrane proteins and RNAs (Lötvall et al., 2014; Théry et al., 2018; Mathieu et al., 2019). Work in the late 1960s first discovered and recorded the presence of vesicles around cells in human plasma (Wolf, 1967). However, the generic term “exosome” was not proposed until 1987 (Johnstone et al., 1987). According to their biogenesis processes, EVs can be classified into three major subtypes: (a) microvesicles, budding from the plasma membrane, with size between 50 and 1000 nm; (b) exosomes: a group of relatively smaller membrane vesicles (30–120 nm) secreted from the endosomal system and formed through the inward budding of multivesicular bodies (MVBs) that transfer molecules into target cells; and (c) apoptotic bodies: derived from fragments of apoptotic cells, sized between 800 and 5,000 nm. As there is no standard production protocol for MSC-EVs, the term “MSC-exosomes” was used to describe exosome-containing products derived from MSCs (Gimona et al., 2021).

The biogenesis of exosomes comprises an endosomal sorting complex required for transport (ESCRT)-dependent mechanism and an ESCRT-independent mechanism (Trajkovic et al., 2008; Kajimoto et al., 2013; Mathieu et al., 2019). The ESCRT-dependent process starts with the inward budding of the bilayer membrane of late endosomes, resulting in the formation of intraluminal vesicles. The bilayer membrane bubble-filled endosome is called an MVB. The maturation of MVBs ends with the excretion of the exosomes into the extracellular space. The ESCRT-independent mechanism can also produce intraluminal vesicles. The biogenesis of microvesicles mainly involves the outward budding of the cell plasma membrane and degradation of the cytoskeleton (Shao et al., 2018). However, other mechanisms may be also involved in the biogenesis of microvesicles, for instance, conversion between ceramide and sphingomyelins (van Niel et al., 2018).

### 3.2 EV isolation and characterization

Differential ultracentrifugation is the most frequently used method for isolation of EVs and remains the “gold standard” (Théry et al., 2018). However, differential ultracentrifugation, which can only be performed for up to about 500 ml of culture medium per run, is unsuitable for large-scale purification (Witwer et al., 2019). Density gradient centrifugation is an improved ultracentrifugation method that produces EVs with higher purity. Although other methods such as serial washes can improve purity, they are associated with low production, risk of structural damage, and contamination (Webber and Clayton, 2013). Therefore, ultracentrifugation may not be an optimal method for therapeutic applications.

Size-based isolation methods include ultrafiltration, size exclusion chromatography, and tangential flow filtration (Li et al., 2017; Willis et al., 2017; Wu et al., 2021b). These methods can produce EVs with comparable or superior purity and/or functional activity to those produced by conventional methods (Busatto et al., 2018; Ludwig et al., 2018); however, pore clogging may occur during the filtration process, resulting in low yield.

Polymer precipitation with polyethylene glycol (PEG) or other polymers could be an effective way to achieve “salting out” of EVs to aggregate (Doyle and Wang, 2019). In clinical applications, PEG or other polymers are considered as contaminants that need to be removed (Doyle and Wang, 2019). Immunoaffinity capture technology, in which antigens are bound to antibodies on the surface of EVs, allows isolation of specific EVs with certain surface proteins.

With the development of the EV research field, various novel strategies have been developed to isolate EVs. However, as there are no standard evaluation metrics for MSC-EV preparation, and no proprietary matrices have been specified, it is difficult to assess the clinical usability of the resulting EVs, as well as the safety of the ingredients used in these EV-harvesting strategies (Reiner et al., 2017).

According to the Minimal Information for Studies of EVs (MISEV) guidelines, the characterization of isolated EVs needs to address morphology, particle size, and surface biomarkers (Théry et al., 2018). The morphology of EVs is observed by scanning electron microscopy, transmission electron microscopy (Théry et al., 2018), electron cryo-microscopy, and atomic force microscopy (Wu et al., 2021b). The size distribution and concentration of EVs are usually determined by nanoparticle tracking analysis, dynamic light scattering, and tunable resistive pulse sensing (An et al., 2021). Common biochemical analysis methods for EVs include western blotting, flow cytometry, and liquid chromatography with mass spectrometry. As the size ranges of exosomes and microvesicles may overlap, these two subtypes cannot be differentiated by size alone.

According to minimal criteria for MSCs suggested by the International Society for Cell and Gene Therapy (ISCT), surface antigens may be the most detectable characteristics that can be transferred to EVs (Dominici et al., 2006). MSC-EVs could be identified based on recently reported MSC-specific surface antigens including CD73, CD90, and CD105, and by the absence of CD14, CD34, and CD11b by Western blot or ELISA (Witwer et al., 2019). The proteomic and lipidomic signatures of MSC-exosomes samples can also be utilized for the identification, characterization, and verification of MSC-exosomes. Previous research revealed that the origin of EV subtypes can be defined by identifying the specific binding affinity, such as cholera toxin B chain (CTB), annexin V (AV), and Shiga toxin B chain (ST) on lipid membrane (Lai and Lim, 2019). And the signature analysis of a specific set of proteins might help in quality control of the preparation and standardization of MSC-exosomes (van Balkom et al., 2019).

## 4 MSC-exosomes in skin regenerative medicine

MSCs that secrete effectors can be loaded into EVs, thus enabling them to exert bioactive effects (Fu et al., 2019). Exosomes are thought to be the critical bioactive EVs responsible for the paracrine effects of MSCs (Joo et al., 2020), which can be secreted from all types of cells in the human body. According to high-throughput exosome studies, exosomes consist of and carry a diverse load of proteins (Toh et al., 2018), lipids (Davidson et al., 2022), metabolites, RNAs (Fan et al., 2018; Zhang et al., 2019b). Almost 350,000 proteins and more than 27,000 mRNAs and 10,000 miRNAs have so far been detected in exosomes, based on data from Vesiclepedia (http://microvesicles.org/, accessed on 1 August 2022).

There is a growing recognition that MSC-derived exosomes are the initial mediators of the therapeutic efficacy of MSCs, maintaining the biological activity and therapeutic effects of progenitor MSCs (Yeo et al., 2013; Brennan et al., 2017; Ramasubramanian et al., 2019; Yin et al., 2019). The mechanisms underlying the therapeutic potential of MSC-exosomes involve transmission of intercellular information through direct binding to surface ligands *via* ligand–receptor interactions (Chen et al., 2018; Theodoraki et al., 2018; Yang et al., 2018) or transfer of genetic information and proteins into acceptor cells through cellular internalization or membrane fusion (Skog et al., 2008; Möller and Lobb, 2020) to alter the biological properties of target cells. Research indicates that luminal vehicles within exosomes are absorbed by recipient cells by three methods: direct membrane fusion (Duan et al., 2021), endocytosis (Gurung et al., 2021), and receptor–ligand interactions (Salunkhe et al., 2020).

MSC exosome-based therapies are thought to circumvent the hurdles faced by MSC-based therapies. The main advantages of MSC-exosomes can be summarized as follows. First, the isolation, concentration, storage, and therapeutic dosage of exosomes are more controllable (Witwer et al., 2013). Second, the phospholipid bilayer structure can merge directly with target cells, releasing functional cargos in the receiving cells. Third, exosome-based therapies can avoid the risks of immune rejection and tumorigenesis (Tan et al., 2021). Therefore, MSC-exosomes, as a promising cell-free therapy, exhibit high potential in wound healing and skin regeneration.

### 4.1 Therapeutic potential of MSC-exosomes in skin tissue regeneration

The pathology of wound healing is regulated by a well-orchestrated processed including inflammation, re-epithelialization, reformation, angiogenesis, and collagen remodeling (Singer and Clark, 1999). MSC-exosomes are believed to have equivalent biological effects to MSCs in tissue regeneration, homeostasis, and wound healing (Yeo et al., 2013; Ramasubramanian et al., 2019; He et al., 2022; Luo et al., 2022; Yan et al., 2022). They can exert their effects on receptor cells by modifying gene expression and protein production. Research efforts have been undertaken to develop MSC-exosomes as therapies in wound healing.

Several studies have examined the effects of MSC-exosomes on the prolonged inflammatory phase in chronic wounds. Anti-inflammatory M2 macrophages have essential roles in promoting cell proliferation and tissue regeneration (Singer and Clark, 1999; Das et al., 2015; Guo et al., 2018). He et al. revealed that bone marrow MSC (BMMSC)-derived exosomes could modulate M2 polarization to promote cutaneous wound healing (He et al., 2019). Adipose tissue-derived stem cell (ADSC)-exosomes have been proven to attenuate macrophage infiltration and reactive oxygen species production (Xu et al., 2020).

Several studies have reported that MSC-exosomes contribute to wound healing with an emphasis on proliferation. Angiogenesis and re-epithelization are important tasks during the proliferation phase of wound healing (Tonnesen et al., 2000; Sorrell et al., 2009). ADSC-exosomes have been reported to promote the proliferation and migration of vascular endothelial cells and sprouting of vascular endothelial tip cells, and to rejuvenate senescent endothelial cells *in vivo* and *in vitro*. Ren et al. reported that the angiogenesis induced by ADSC-exosomes in human umbilical vein endothelial cells was associated with the upregulation of platelet-derived growth factors, VEGFA, EGF, and FGF *via* AKT and ERK signaling pathways (Ren et al., 2019). In a mouse model of a full-thickness skin defect, exosomes from fetal dermal MSCs could accelerate wound healing by activating Notch signaling (Wang et al., 2019a). In an ischemic disease model, miRNA-31 in ADSC-exosomes targeted factor inhibiting HIF-1 (FIH-1) in recipient cells to promote angiogenesis (Zhao et al., 2017; Bian et al., 2019). In addition, preclinical studies in mouse injury models have shown that BMMSC-exosomes accelerate wound healing by promoting collagen synthesis and angiogenesis (El-Tookhy et al., 2017; He et al., 2019).

Human-induced pluripotent stem cells can be generated from a variety of adult cell types after genetic modification. Exosomes from induced pluripotent stem cells (hiPSC)-MSCs can accelerate cutaneous wound healing by promoting vascularization and fibroblast proliferation in rats. After an injury, wounds treated with hiPSC-MSC-exosomes showed improved re-epithelialization and formation of skin appendices, including sebaceous glands and hair follicles. Increasing expression of CD-31 and α-SMA indicates proliferation of endothelial cells and smooth muscle cells that are closely related to vascularization (Zhang et al., 2015). Notably, hiPSC-MSC-exosomes were also found to enhance collagen synthesis and fibroblast proliferation in a dose-dependent manner of human umbilical vein endothelial cells *in vitro*. The proliferation of human keratinocytes (HaCaT cells) and human dermal fibroblasts was also found to increase significantly after treatment with hiPSC-MSC-exosomes (Kim et al., 2018).

MSC-derived exosomes also have prominent regenerative effects on collagen deposition and can reduce scar deposition. Data suggest that exosomes from human ADSCs can promote fibroblast proliferation and migration through the PI3K/Akt signaling pathways following subcutaneous injection of umbilical cord blood (UCB)-exosomes at full-thickness cutaneous wounds on the backs of mice (Zhang et al., 2018). Another study confirmed the beneficial effects of exosomes from human umbilical cord blood plasma (hUC-MSCs) on wound healing. Levels of collagen I, collagen III, and α-SMA were downregulated after treatment with hUC-MSC-derived exosomes, and expression of myofibroblast-related protein was decreased. In addition, hUC-MSCs have been found to inhibit the activation of the TGF-β1/Smad2/3 signaling pathway. These data indicate that hUC-MSC-derived exosomes could inhibit the transition of fibroblasts to myofibroblasts (Hu et al., 2020).

However, exosome therapeutics for wound healing still face challenges regarding their clinical applications, including poor targeting, a rapid clearance rate, and a relatively short half-life in the complex microenvironment of the wound (Liu et al., 2017). Quantitative analysis of exosome-internalized skin cells show a time-dependent decrease 24 h after injection (Hu et al., 2018). Furthermore, the functions of these cells may have been compromised by the time-consuming harvesting and passage processes and the low survival rate. For these reasons, tissue engineering technology has been introduced to modify the therapeutic potential of natural exosomes, enabling the design of exosomes higher stability and greater therapeutic capability in accelerating wound healing and skin regeneration.

### 4.2 Challenges in the development of MSC-exosomes-based therapies

Although several preclinical studies have shown promising result, some challenges still hinder the development of exosome-based treatments (Liu et al., 2017; Hu et al., 2018). In clinical settings, the drawbacks of exosome-based therapy may outweigh the advantages. Challenges in achieving a therapeutic effect in clinical settings are found throughout the whole process of MSC-exosome preparation.

To date, various studies are evaluating the biodistribution of exosomes obtained from different sources and used for treatment in several disease models. The underlying mechanism of action (MoA) of MSC-exosomes in each stage of wound healing needs further elucidating. To achieve the therapeutic effect, MSC-exosomes need to present on the target site in a spatiotemporal manner. However, no specific markers are yet available to specifically label and track the biodistribution of MSC-exosomes (He et al., 2019). Moreover, to elucidate the MoA of the therapeutic MSC-exosomes is often implemented by gene editing of the donor MSCs to down-regulated the candidate RNAs or proteins of MSC-exosomes, which may eventually lead to major alterations of MSC-exosomes products (Théry et al., 2018).

The variability of parental MSC sources, the processes for isolation, purification, and identification of exosomes, and the need for a standardized definition of MSC-exosomes need to be addressed (Witwer et al., 2019). In addition, the therapeutic efficacy of MSC-exosomes may be affected by local conditions, the delivery route, and the time window for intervention. For clinical applications of MSC-exosomes, xenogenic components should be avoided during the preparation process. Non-MSC-exosomes derived from the culture media may exist (Witwer et al., 2019).

**FIGURE 1:**
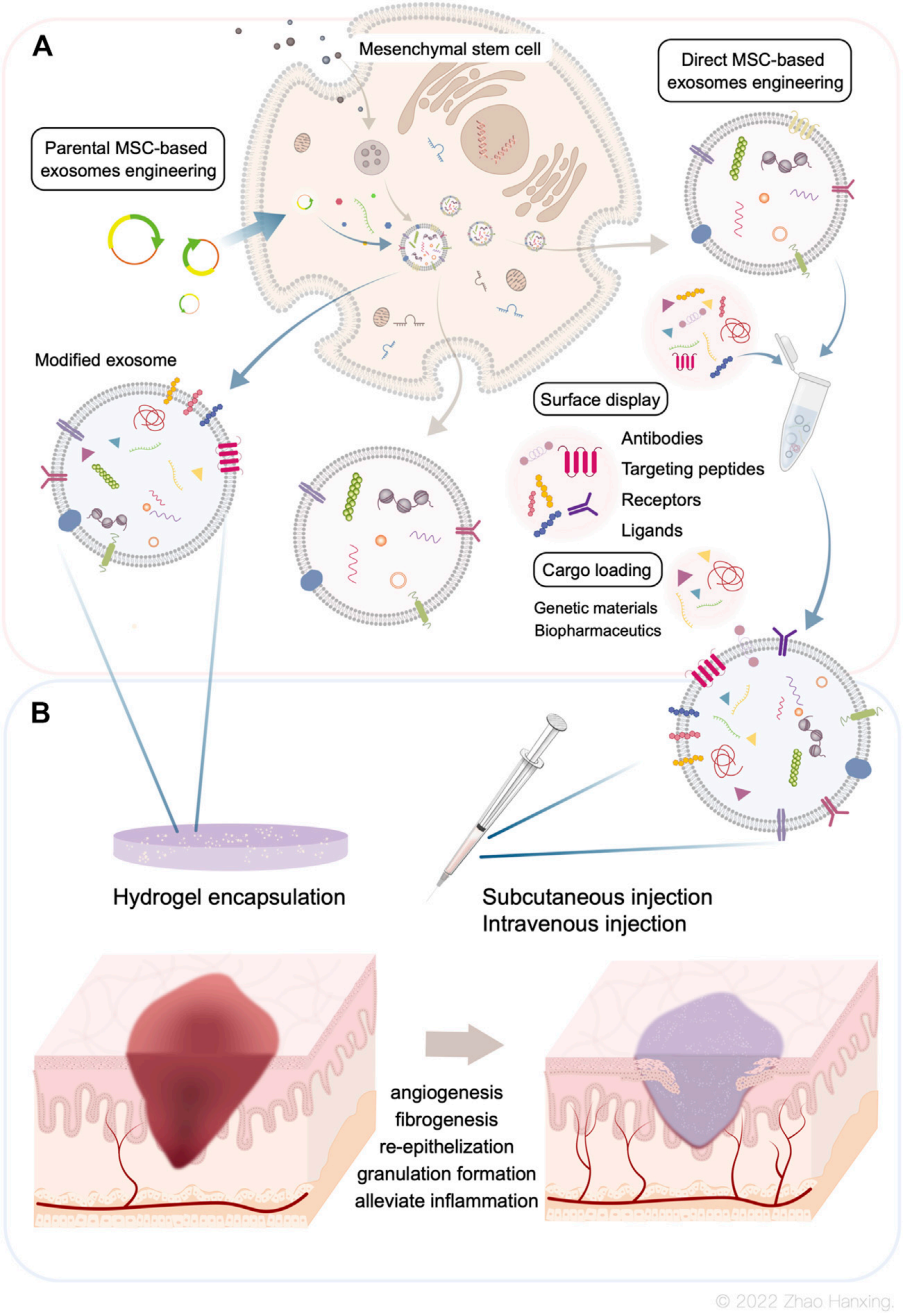
**(A)** Biogenesis of Mesenchymal stem cell-derived exosomes. The bioengineering modifications include genetic modification of MSCs and direct modification of exosomes. **(B)** Current administration procedures of modified exosomes are co-administration with hydrogels and administered by intravenous, subcutaneous, or intraperitoneal injections. Modified MSC-exosomes could accelerate skin wound repair by promoting angiogenesis, fibrogenesis, re-epithelization, and granulation tissue formation, as well as alleviate inflammation.

After administration, the biological effects of MSC-exosomes should be elicited only after they have been engulfed by the target cells. Otherwise, MSC-exosomes would be depleted by immune cells in the circulation. Exosomes are often administered intravenously, subcutaneously, or intraperitoneally (Figure 1). There are also drawbacks also associated with the administration of the exosomes. Systemic administration of exosomes may cause an instant inflammatory reaction and rapid clearance from the blood circulation, resulting in insufficient residence time and homing. The half-life of local administration of exosomes may be even shorter with low bioavailability owing to flushing by body fluid, resulting in a short retention rate of exosomes (Agrahari et al., 2016; Riau et al., 2019). Moreover, therapeutic efficacy may differ between exosomes harvested from *in vitro* MSC culture and exosomes secreted by MSCs after administration *in vivo*.

## 5 Bioengineering modified MSC-exosomes

**FIGURE 2:**
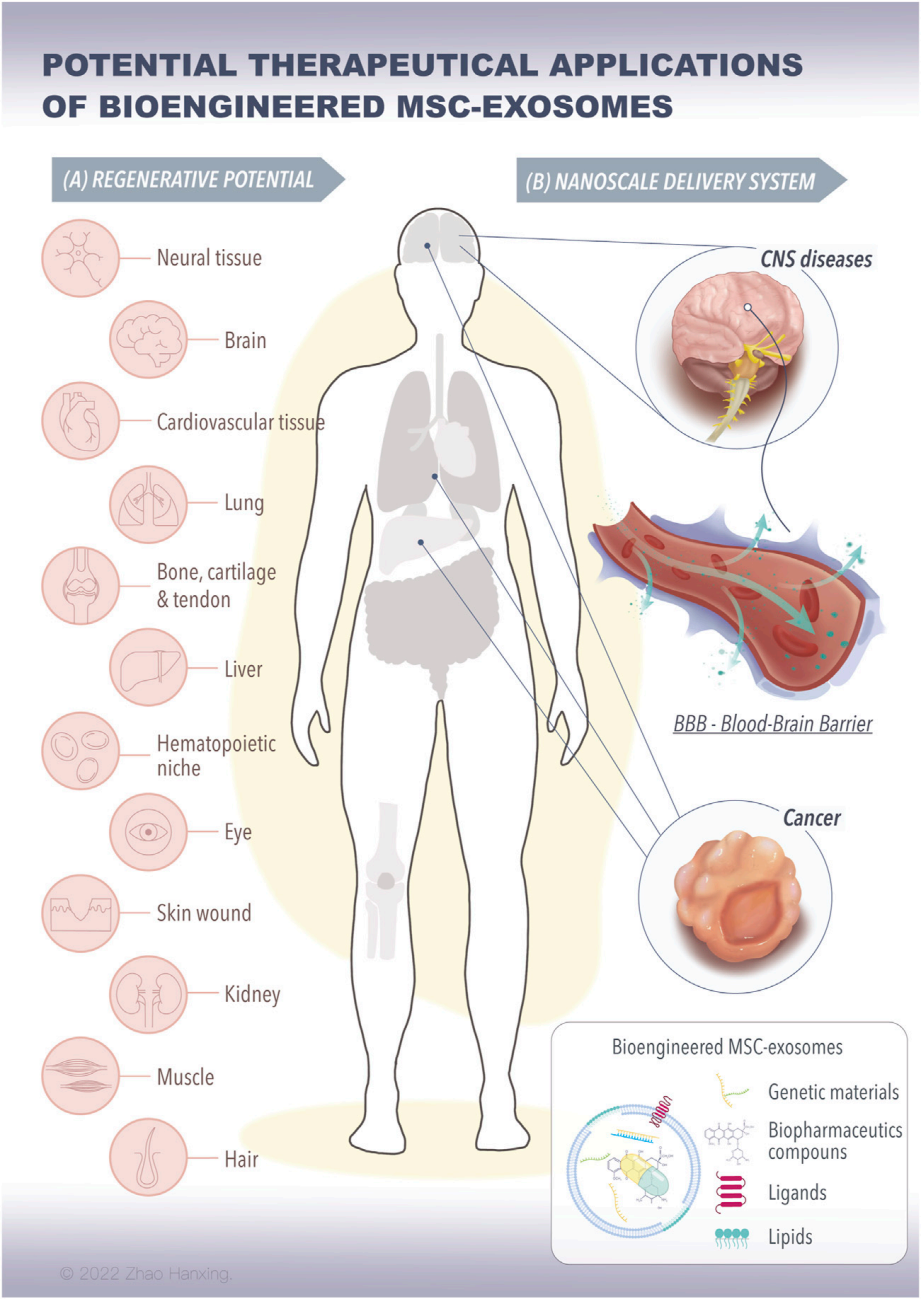
The potential therapeutical applications of bioengineered MSC-exosomes for tissue regeneration and nanoscale delivery system. **(A)** The bioengineered MSC-exosomes loading with specific signaling molecules, such as proteins, RNAs, and nanoparticles can achieve repair and regenerative effects on various tissues. **(B)** The bioengineered MSC-exosomes can cross the BBB in the brain or to the tumor for the treatment of CNS diseases and cancer. (MSC, mesenchymal stem cells, BBB, blood-brain barrier, CNS, central nervous system).

To solve the problems of low effective concentration and limited therapeutic effect, researchers have introduced bioengineering methods into exosome-based therapy. This has resulted in the design of highly specialized bioengineering-modified MSC-exosomes. The features of modified MSC-exosomes are as follows: targeted binding to a specific cell type or tissue; the capacity for loading molecules, drugs, proteins, or genetic information into exosomes or on their surface; and enrichment of an endogenous molecule in the lumen of exosomes or on their surface (Kojima et al., 2018). Current bioengineering methods fall into two main categories: parental MSC-based exosome engineering and direct MSC-exosome engineering (Figure 1A). Several studies have shown the therapeutical roles of bioengineered MSC-exosomes in tissue regeneration and the treatment of CNS disease and cancer (Figure 2) (Haney et al., 2015; Samadikuchaksaraei et al., 2016; Togliatto et al., 2016; Gilligan and Dwyer, 2017; Tao et al., 2017; Yuan et al., 2017; Li et al., 2018b; Liao et al., 2018; Luo et al., 2019; Mathew et al., 2019; Sisa et al., 2019; Kaminski et al., 2020; Xuan et al., 2020; Nuzzi et al., 2021; Sandonà et al., 2021; Yao et al., 2021; Dong et al., 2022; Kurniawati et al., 2022; Miyasaki et al., 2022; Thomas et al., 2022; Rong et al., 2023).

### 5.1 Parental MSC-based exosome engineering

The genetic engineering of parental MSCs produces specific exosomes loaded with functional surface display molecules to specificically label MSC-exosomes or increase exosome accumulation. The most common procedure for encapsulating a protein at the surface of exosomes is the use of an exosome signal peptide such as lysosome-associated membrane protein 2b (LAMP2b) (Alvarez-Erviti et al., 2011). The LAMP2b membrane protein combined with a neuron-targeted short peptide of rabies virus glycoprotein (RVG) is expressed. RVG displayed on the surface may lead to the accumulation of exosomes and enhance exosome delivery (Alvarez-Erviti et al., 2011; Barile and Vassalli, 2017). Vesicular stomatitis virus glycoprotein is an exosome-anchored transmembrane protein containing ectoplasmic, transmembrane helix, and cytoplasmic domains (Meyer et al., 2017). Specific proteins can replace the ectoplasmic and cytoplasmic domains without altering the structures of the exosome membrane. Other documented exosome surface proteins include tetraspanins (CD63, CD9, CD81) (Mathieu et al., 2021; Shi et al., 2021), glycosylphosphatidylinositol (GPI)-anchored cadherin (Nakamura et al., 2020), platelet-derived growth factor receptors (Ohno et al., 2013), and lactadherin (C1C2 domain) (Tian et al., 2021), which could be used for similar purposes.

The EXOtic device can be used to carry mRNAs into parental MSCs to improve exosome production, deliver mRNAs into recipient cells, and promote cell-to-cell communication (Kojima et al., 2018). CRISPR/Cas9 technologies are employed for *in vivo* gene editing. For example, CRISPR/Cas9 has been used to knock down specific gene sequences in human umbilical MSCs to enhance therapeutic effects (Shao et al., 2020). Such strategies have great potential for gene manipulation in exosome-based therapy and are awaiting further investigation. Transfection has been used to incorporate small RNAs and endogenous molecules into exosomes *via* loading into parental MSCs. Parental MSCs co-transfected with plasmids encoding production enhancement genes may increase exosome production by 15- to 40-fold as determined by measuring a luminescent reporter (Kojima et al., 2018).

Genetic modification of parental MSCs seems feasible for improving protein surface display, increasing yield of exosomes, loading specific mRNAs into the exosomes, and increasing exosome uptake by recipient cells without any alteration in exosome structures. However, alteration of genetic information may not be suitable for use in exosomes for clinical applications.

### 5.2 Direct MSC-exosome engineering

Direct exogenous loading approaches after exosome isolation include co-incubation, electroporation, and sonication; other methods such as freeze-thaw and extrusion are rarely used (Figure 1).

Co-incubation is a frequently used strategy to maintain the integrity of the exosome membrane and is especially suitable for loading of hydrophobic molecules such as drugs and RNAs (Xiao et al., 2022; Zhuang et al., 2022). Electroporation is a promising strategy for loading small-molecule drugs or nucleotides into exosomes by creating small-transit pores on the phospholipid bilayer (Lv et al., 2020; Xiong et al., 2022). The membrane gap is automatically restored without altering the morphology of the exosomes. Given that large compounds cannot easily be encapsulated in exosomes, the electroporation approach is particularly suitable for loading nucleotides (Luan et al., 2017). Sonication is also a popular method for exosome engineering (Haney et al., 2015). Exogenous cargo diffuses into the exosomes by passing through the membrane breaks caused by a probe’s mechanical force. Although the loading efficiency of the sonication method is higher than those of co-incubation and electroporation, this approach has some important shortcomings. For instance, the high power of sonication may cause irreversible deformation of exosomes and may disrupt the integrity of exosomes (Haney et al., 2015; Luan et al., 2017).

Extrusion can be used to load exogenous cargos such as gold nanoparticles into exosomes (Khongkow et al., 2019; Van Deun et al., 2020). Freeze–thaw methods have been used to load water-soluble molecules into exosomes by forming ice crystals within lipid membranes that temporarily break the integrity of the exosome membrane (Haney et al., 2015; Hajipour et al., 2021). According to a previous study, extrusion and freeze-thaw methods have notable drawbacks, including low efficiency, low throughput, and the risk of altering the membrane stability of exosomes, resulting in unpredictable side effects (Fuhrmann et al., 2015; Haney et al., 2015; Van Deun et al., 2020). Therefore, such methods are not conventionally used for loading cargo into exosomes.

Exosomes can be modified by using chemical engineering strategies such as click chemistry and metabolic glycoengineering to conjugate ligands to the exosome surface, enabling regulation of the directional migration and targeting of exosomes (You et al., 2021). The advantage of this method is that the highly effective reaction can maintain the size of exosomes without any undesirable side reaction related to the association between exosomes and recipient cells (Smyth et al., 2014; Yoon et al., 2017).

Engineered hybrid exosomes are emerging as an alternative strategy for encapsulating and transferring large nucleic acids such as DNA to target cells and organs and exert therapeutic effects (Lin et al., 2018). Fusing exosomes with synthetic liposomes modifies and tunes the exosome interface to decrease immunogenicity, increase colloidal stability, and improve the half-life in circulation (Lin et al., 2018). Composite carriers enable the efficiency of drug loading to be improved while retaining the function of exosomes. However, there have been few comprehensive evaluations of hybrid exosomes. Hybrid exosomes have been reported to show cytotoxicity slightly greater than that of pure exosomes, as demonstrated by their effects on cell proliferation, which was attributed to the conjugation of the nanoparticles (Chen et al., 2020). As the incubation time or the concentration of hybrid exosomes increased, the viability of progenitor cells decreased.

In summary, various therapeutic molecules and nanoparticles can be efficiently transferred to exosomes using the abovementioned modification procedures. However, these methods require repeated purification and centrifugation using detergents, enzymes or other methods, which might disrupt the integrity of exosomes or reduce bioactivity (Levy et al., 2022). Therefore, novel bioengineering procedures are needed to compensate for these limitations.

### 5.3 Co-administration with hydrogels

Deepening understanding of exosome-based therapies has spurred research combining biomaterials to formulate sustained-release therapeutic systems. Improving the ability of MSC-exosomes to accelerate wound healing is the driving force behind the research integrating biomaterials and bioengineering. Ideal biomaterials can be used to maximize the therapeutic functions of exosomes as wound dressings or scaffolds by increasing their durability and stability.

A variety of biomaterials, including membranes, electrospun nanofibers, colloidal nanoparticles, and hydrogels, have been used to facilitate the controlled release of bioactive molecules for skin regeneration (Liang et al., 2019; Bai et al., 2022; Hu et al., 2022). Among them, hydrogels have recently attracted attention for applications as delivery systems and scaffold dressings with multifunctional properties such as antibacterial activity, hemostatic ability, tissue adhesion, anti-ultraviolet activity, injectability, and self-healing (Lokhande et al., 2018; Safari et al., 2022). Hydrogels can mimic natural extracellular matrix (ECM) and provide a three-dimensional framework to support growth and proliferation of loaded cells and regulate biomolecule activation (Riha et al., 2021). Moreover, three-dimensional hydrophilic polymer networks can maintain moisture at the wound site.

The combination of hydrogels and exosomes plays a crucial part in adjusting the wound inflammation microenvironment, promoting vascularization, enhancing re-epithelialization, and accelerating wound healing (Qian et al., 2020; Zhang et al., 2021a; Kudinov et al., 2021; Geng et al., 2022). Furthermore, a comparative study has demonstrated a synergistic effect of hydrogel–exosome complexes compared with exosome therapy alone (Zhou et al., 2022).

In summary, although several studies have shown great potential for MSC-exosomes in clinical applications, many unresolved issues still limit their practical use. Before exosomes are introduced into clinical settings, safety concerns, cytotoxicity, and reproducibility of production need to be solved (Luan et al., 2017; Murphy et al., 2019). The development of bioengineering methodologies for exosome-based therapy could provide a means of bridging the gap.

## 6 Applications of bioengineering-modified MSC-derived exosomes in skin wound healing and regeneration

In this section, we review the therapeutic applications of modified MSC-exosomes in wound healing and skin regeneration and the improvements in regenerative efficiency that have been achieved compared with MSC-derived exosomes. As mentioned in the previous discussion on modified exosomes and bioengineering technology, there are two current trends for implementing modified MSC-exosomes in wound healing and skin regeneration.

### 6.1 Bioengineering the properties of MSC-exosomes

One solution is to bioengineer the properties of exosomes, such as their cargos or surface molecular functions, before administration (Table 1).

**TABLE 1:** Summary of research concerning engineered exosomes in wound repair.

Exosome type	Model	Engineering strategy	Function	Reference
BMSC-exosomes	skin wound mouse model	Loading miRNA-542-3p into exosomes by electroporation	promote cellular proliferation, collagen deposition, neovascularization, and accelerated wound closure	Xiong et al. (2022)
BMSC-exosomes	calvarial defect rat model	BMSC-exosomes incubated with Fe3O4 nanoparticles with or without a magnetic field	Enhance angiogenesis and osteogenesis	Wu et al. (2021a)
UCMSC-exosomes	*In vitro* study	three-dimensional coculture of UCMSCs and endothelial cells under hypoxic conditions	promoting proliferation and inhibiting apoptosis	Zhang et al. (2021b)
Engineered miR-31 exosomes	chronic diabetic wounds	transfected miR-31-5p lentiviral vector into HEK293 cells	enhancing angiogenesis, fibrogenesis and reepithelization	Huang et al. (2021)
engineered exosomes-derived from monocytic cells	HUVEC	Exosomes treated with immunomodulating compounds	facilitate HUVECs tube formation and enhance skin cell proliferation and migration	Su et al. (2022)
BMSC-exosomes	diabetic (db/db) mice	BMSCs transfected to overexpress long non-coding RNA HOX transcript antisense RNA	promote angiogenesis and wound healing	Born et al. (2022)
miR-29a-modified hADMSC-exosomes	thermal mouse model	Transfected miR-29a mimics into hADSCs	reduce excessive scar formation	Yuan et al. (2021)
TSG-6 modified MSC-exosomes	mouse full-thickness wound model	overexpression and knockdown of TSG-6 lentivirus infection into hBMSCs	suppressed scar formation *via* reducing inflammation and inhibiting collagen deposition	Jiang et al. (2020)
MSC-EV	*In vitro*: HUVECs	thrombin preconditioning regimen	enhanced proliferation, the migration and tube formation of HUVECs *in vitro*, and cutaneous wound healing *in vivo*	Sung et al. (2019)
	*In vivo*: cutaneous wound healing			

Abbreviations: Exo: exosome; HUMSC: human umbilical mesenchymal stem cell; ADMSC: adipose-derived mesenchymal stem cell; SMSC: synovial mesenchymal stem cells; MSC: mesenchymal stem cell; EV: extracellular vesicles.

The RNA cargo of MSC exosomes is considered a pivotal factor in eliciting biological functions to target cells and has raised great attention. Previous mechanistic studies have shown that exosome-derived RNAs play a crucial role in immunomodulatory capacity (He et al., 2019; Zhao et al., 2021; Wang et al., 2022), stimulate the differentiation of fibroblasts, keratinocytes, and endothelial cells, promote angiogenesis (Liang et al., 2016), and prohibit tissue hyperplasia and scar formation (Fang et al., 2016). Such abilities can be enhanced after bioengineering modification. For example, modified exosomes derived from bone marrow MSCs (BMMSCs) enriched with miRNA-542-3p could enhance proliferation, migration, and angiogenesis of human skin fibroblasts/human dermal microvascular endothelial cells *in vitro* and *in vivo* (Xiong et al., 2022). The potential therapeutic value of exosomes loaded with circular RNAs has been studied in full-thickness skin wound repair in diabetic rats. The MSC-exosomes enriched with mmu_circ_0000250 could efficiently regulate miR-128-3p and the expression of SIRT1 to control inflammation (Shi et al., 2020b). Another study modified MSCs to generate exosomes enriched for long ncRNA H19, leading to the regulation of the PI3K/AKT signaling pathway and suppression of inflammatory responses and apoptosis in a diabetic foot ulcer mouse model (Li et al., 2020). Nanoparticles can be loaded into the exosomes to promote angiogenesis. BMSCs-exosomes loaded with magnetic Fe_3_O_4_ nanoparticles, with upregulation of exosome miR-1260a, were confirmed to enhance angiogenesis (Wu et al., 2021a).

Deep sequencing of MSC exosomal RNA revealed that MSC excretes exosomal miRNAs in a selective manner, many of which are precursor miRNAs incapable of protein-coding (Chen et al., 2010; Pritchard et al., 2012). In addition, a prerequisite for RNA-based MoA is that the exosome-derived miRNAs reach a therapeutic dose. However, the amount of miRNAs in standard preparation is approximately 60 ng per 100 μg exosome. Leaving aside the fact that the distribution of a specific miRNA in an exosome is about 1:100 (Chevillet et al., 2014; Albanese et al., 2021). According to the analysis of Toh WS et al., MSC-exosome-derived miRNAs are unable to achieve the therapeutic effect as their low concentration in single exosome, immature status, and the absence of RNA-induced silencing complex (RISC) (Toh et al., 2018).

The protein-mediated mechanism of MSC-exosomes in wound healing has raised attention (Sung et al., 2019; Bari et al., 2020; Kudinov et al., 2021; Camões et al., 2022; Zhang et al., 2022). Comprehensive proteomic analysis showed that the proteins TGF-β, ITGA1-3/5, IL-6, CDC151, S100A10, and Wnt5α, as being enriched in MSC-exosomes in 3D culture, were confirmed to be associated with wound healing (Camões et al., 2022). In infected burn injury, PGE2, IL-6, IL-8, or IFN-γ, IL-10, growth factors, and chemokines secreted by placental MSC secretome were found to accelerate dermal fibroblast and keratinocyte migration and proliferation, stimulate angiogenesis, and reduce scarring (Kudinov et al., 2021). Angiogenic protein cargos, including angiogenin, angiopoietin-1, HGF, and vascular endothelial growth factor (VEGF) in thrombin preconditioning MSC-exosomes were enhanced compared with that of naïve MSC-exosomes, thus boosting angiogenesis (Sung et al., 2019). As with RNA-based MoA, the proteins should be in therapeutic doses to elicit their therapeutic effect.

### 6.2 Hydrogel encapsulation of MSC-exosomes

Another bioengineering strategy combines exosomes and hydrogels to exert synergistic functions after administration (Table 2) (Wang et al., 2019b). Hydrogels act as three-dimensional porous scaffolds to provide a favorable environment for cell proliferation and ECM remodeling (Yang et al., 2020). A complex of exosomes and hydrogels can also act as a sustainable release system for an extended period and exert lasting curative functions. The flexible morphology of biomaterials enables the construction of more variable forms of exosomes (Wang et al., 2019c).

**TABLE 2:** Summary of research concerning exosome-loaded scaffold in wound repair.

Exosome type	Model	Scaffold	Function	Reference	
fibrosarcoma cell line HT1080-Exos	*In vitro* study	A thermo-responsive polymer of poly(N-vinyl caprolactam) (PNVCL) for encapsulation of exosomes	facilitate thrombus degradation and healing of endothelium lining	Das et al. (2022)	
GMSCs-Exos	diabetic rats	GMSC-Derived Exosomes Combined with a Chitosan/Silk Hydrogel Sponge	promoting the re-epithelialization, deposition and remodeling of collagen and enhancing angiogenesis and neuronal ingrowth	Shi et al. (2017)	
hADMSCs-Exos	*In vitro* study	Elastomeric Scaffolds (polycaprolactone)	increasing the wound healing properties and collagen type I and vitronectin of the MSC, and improving the M2 phenotype of the macrophages	Chachques et al. (2021)	
BMSC-exosomes	chronic diabetic wound healing	BMSC-exosomes-loaded carboxyethyl chitosan (KimParaiso et al., 2011)-dialdehyde carboxymethyl cellulose (DCMC) hydrogel (MSC-exosomes@CEC-DCMC HG)	adjusted the wound inflammation microenvironment, promoted neovascularization, and accelerated wound healing in type 1 diabetic rats	Geng et al. (2022)	
ADMSC-exosomes	rat full-thickness skin injury model	AMSC-exosomes-loaded β-chitin nanofiber hydrogel	acceleration rate of wound closure	Liu et al. (2022)	

Abbreviations: Exo: exosome; ADMSC: adipose-derived mesenchymal stem cell; GMSC: gingival mesenchymal stem cell.

MSC-exosomes encapsulated by biomaterials could avoid being rapidly released into the bloodstream and exert their therapeutic effect in a dose-dependent manner at designated sites. Hydrogels can be used as loading vehicles to enhance exosome retention rates and achieve synergistic therapeutic efficacy (Wang et al., 2019b; Das et al., 2022). A thermoactivated polymer has been shown to enable sustained administration of exosomes, maintaining 65% of the initial number of exosomes after 25 days (Das et al., 2022). This study also found no cytotoxic effects on cells in contact with the hydrogels or when cells were embedded in the hydrogel constructs. Surface modification with nanoparticles increases the production of exosomes. Bioavailable nanoparticles based on iron oxide with poly(lactic-co-glycolic acid) were designed to improve the yield of exosomes by stimulating MVB formation (Park et al., 2020).

Biomaterials may also combine engineered exosomes to improve the therapeutic efficiency of exosomes in wound healing (Table 3). As three-dimensional porous scaffolds, hydrogels provide exosomes with a favorable environment that promotes cell proliferation and ECM remodeling and have thus gained more research attention. Furthermore, the transformation of biomaterials provides a greater scope for applications of exosomes (Wang et al., 2019c; Yang et al., 2020). An *in vivo* study showed that incorporating gingival-MSC-derived exosomes into a chitosan/silk-based hydrogel sponge could facilitate wound healing by promoting re-epithelialization, angiogenesis, and neuronal ingrowth, as well as collagen remodeling (Shi et al., 2017). In a study by Wang et al., an injectable methylcellulose-chitosan hydrogel loaded with exosomes derived from human placental MSCs was constructed to synergistically promote angiogenesis and inhibit apoptosis (Wang et al., 2019b). The modified exosome hydrogel complex forms a suitable injectable wound dressing with appropriate gelation time, mechanical properties, and high self-healing efficiency. In a diabetic wound-infected model, BMSC-exosomes were loaded in an antibacterial hydrogel and enabled the wound inflammation microenvironment to be adjusted, as well as promoting neovascularization and accelerating wound healing (Geng et al., 2022).

**TABLE 3:** Summary of research concerning engineering exosomes-loaded scaffold in wound repair.

Exosome type	Model	Engineering strategy	Function	Reference
hADSCs-Exos	a diabetic wound festers model	reductive 2D COFs as a nanocarrier to immobilize engineering exosomes (E-Exos) collected from TNF-α-treated mesenchymal stem cells (MSCs) under hypoxia	suppressing oxidative injury and tissue inflammation, promoting angiogenesis and eradicating bacterial infection	Sun et al. (2022)
HUMSCs-Exos	*P. aeruginosa* infected mouse skin wound defect model	an asymmetric wettable dressing with a composite of exosomes and silver nanoparticles (CTS-SF/SA/Ag Exo dressing)	CTS-SF/SA/Ag-Exo dressing enhanced wound healing by accelerating collagen deposition, angiogenesis and nerve repair	Qian et al. (2020)
ADMSC-exosomes	Chronic full-thickness non-healing diabetic wound	Engineering Bioactive Self-Healing Antibacterial Exosomes Hydrogel	enhanced wound closure rates, fast angiogenesis, re-epithelization and collagen deposition within the wound site	Wang et al. (2019d)
SMSCs-126-Exos	diabetic chronic wound	SMSCs-126-Exos with hydroxyapatite/chitosan (HAP-CS) composite hydrogels (HAP-CS-SMSCs-126-Exos)	promote wound surface re-epithelialization, accelerate angiogenesis, and expedite collagen maturity	Li et al. (2016)

Abbreviations: Exo: exosome; COF: covalent organic framework; HUMSC: human umbilical mesenchymal stem cell; ADMSC: adipose-derived mesenchymal stem cell; SMSC: Synovial mesenchymal stem cells.

### 6.3 Clinical trials of MSC-EVs for wound healing and skin regeneration

Overall, bioengineering technologies have been implemented in exosome-based therapy to make MSC-derived exosomes more effective in treating chronic wounds. Previous valuable research findings have expanded the scope of applications of exosomes in treatment of cutaneous wounds and established a theoretical basis for clinical trials of bioengineered MSC- exosomes.

## 7 Prospects and conclusion

Skin wound healing is a complex multi-stage biological process. Chronic wounds characterized by poor vascularization and prolonged inflammation still threaten patients’ health and quality of life. Traditional treatments for skin damage are time-consuming and costly, and existing therapies have little therapeutic effect in promoting chronic wound healing. Therefore, new therapeutic options are needed.

Over the past decades, therapies based on MSCs with multi-lineage potential have shown impressive therapeutic efficacy in regenerative medicine (Pittenger et al., 1999). However, significant challenges regarding immunocompatibility, stability, migration capacity, and pluripotent development still need to be addressed (Jiang et al., 2002). Current opinion is that MSCs exert their therapeutic effects mainly *via* their paracrine activity, mostly through the release of EVs (Herrera et al., 2010; Lai et al., 2010; Lener et al., 2015). As the main bioactive factor of the MSC secretome, MSC-exosomes are reported to have similar therapeutic effects to MSCs that transfer genetic information or biologically active molecules into target cells (Zhang et al., 2014).

In light of these properties, considerable research efforts have been put into MSC-exosomes therapies. In preclinical models, MSC-exosomes have been shown to have several advantages compared with MSCs, including minimal immune rejection and tumorigenesis risk (Zhang et al., 2014) and no side effects *in vivo* (Kordelas et al., 2014). However, before translation of MSC-exosome-based therapeutics into clinical settings, several issues need to be addressed.

The challenges include the manufacturing process of MSC-exosomes, such as characterization, isolation, purification, clinical safety of donor cells and recipient cells, and the manufacturing process. In addition, quality control hinders the translation of MSC-exosome-based therapies into clinical applications.

Microvesicles and exosomes have some common characteristics; for instance, they both have a bilateral phospholipid membranous structure and contain specific proteins, lipid, as well as RNAs, and their size ranges overlap; thus, current isolation procedures cannot discriminate different EV subtypes (Gould and Raposo, 2013; Giebel et al., 2017). Moreover, different manufacturing processes and apparently homogenous EV-releasing MSCs can result in different EV subtypes (Börger et al., 2017).

Currently, the definition of MSC-exosomes is based on the ISCT minimal criteria and the MISEV recommendations (Dominici et al., 2006; Théry et al., 2018). However, both of these sources of guidance are insufficient to distinguish MSC-exosomes from non-MSC-exosomes, and there is no process for functional assessment of MSC-exosomes. Therefore, in this review, the term “MSC-exosome” was used to describe exosome-containing products derived from MSCs. Another challenge during the application of MSC-exosomes in clinical studies is the lack of a standardized manufacturing process to produce the final product. Quality tests for batch size, purity, potency, reproducibility, safety, and storage stability of the MSC-exosome products need to be established. Currently, there is no optimal isolation method to achieve high purity without compromising the integrity, yield, or functionality of the product (Reiner et al., 2017). Finally, storage and transport conditions of MSC-exosome products must also be addressed. Recently, several studies on the storage conditions and biological activity of the native and cargo-loading MSC-exosomes have carried out the ideal storage conditions (Levy et al., 2022). These results may suggest that lyophilization and storage at room temperature can preserve the enhanced bioactivity of cargo-loading exosomes. In this case, additional steps are required to verify the producibility of the MSC-exosomes as a clinical product.

With advances in engineering and biotechnology (Alvarez-Erviti et al., 2011; Haney et al., 2015; You et al., 2021; Xiao et al., 2022; Zhuang et al., 2022), MSC-exosomes produced under given conditions may mitigate some of the shortcomings of natural MSC-exosomes mentioned above. MSC-exosomes, as natural carriers of genetic information and proteins, can potentially be modified as targeted therapeutic delivery systems by genetic editing of parental MSCs (Alvarez-Erviti et al., 2011) or by direct modification of MSC-exosomes (Haney et al., 2015; You et al., 2021; Xiao et al., 2022; Zhuang et al., 2022). Comparing with synthetic nanoparticles delivery system, EVs delivery RNA and protein cargos to cells shown higher efficacy (Murphy et al., 2021). However, the underlying MoA, feasibility, efficiency and producibility of engineering MSC-exosomes products need to be confirmed.

Taken together, the studies reviewed here suggest that MSC-exosomes could provide an alternative option for wound healing. However, more research is required to make modified MSC-exosomes more widely available and suitable for clinical applications in wound healing and skin regeneration.

## Abbreviations

MSCs, mesenchymal stem cells; MSC-exosomes, MSC-derived exosomes; EVs, extracellular vehicles; MiRNAs, microRNAs; NcRNAs, non-coding RNAs; LAMP2b, lysosome-associated membrane protein 2b; RVG, rabies virus glycoprotein; VSVG, Vesicular stomatitis virus glycoprotein; GPI, glycosylphosphatidylinositol; PDGFRs, platelet-derived growth factor receptors; hUMSCs, human umbilical mesenchymal stem cells; MGE, metabolic glycoengineering; BMSCs, bone marrow mesenchymal stem cells; CircRNAs, circular RNAs; DFU, diabetic foot ulcer; NPs, nanoparticles; PLGA, poly (lactic-co-glycolic acid); ECM, extracellular matrix; GMSC, gingival mesenchymal stem cells; ADMSC, adipose-derived mesenchymal stem cell; SMSC, Synovial mesenchymal stem cells.
